# A TRPM2-Driven Signalling Cycle Orchestrates Abnormal Inter-Organelle Crosstalk in Cardiovascular and Metabolic Diseases

**DOI:** 10.3390/biom15081193

**Published:** 2025-08-19

**Authors:** Maali AlAhmad, Esra Elhashmi Shitaw, Asipu Sivaprasadarao

**Affiliations:** 1 Department of Biological Sciences, College of Science, Kuwait University, Alshadadiya, P.O. Box 5969, Safat 130602, Kuwait; maali.alahmad@ku.edu.kw; 2 School of Biomedical Sciences, Faculty of Biological Sciences, University of Leeds, Leeds LS29JT, UK; fy14ees@leeds.ac.uk

**Keywords:** oxidative stress, reactive oxygen species, TRPM2, NOX2, PARP, calcium, zinc, mitochondria, lysosomes, inter-organelle communication, ageing, cardiovascular diseases, metabolic diseases, metabolic syndrome

## Abstract

Cardiovascular and metabolic disorders significantly reduce healthspan and lifespan, with oxidative stress being a major contributing factor. Oxidative stress, marked by elevated reactive oxygen species (ROS), disrupts cellular and systemic functions. One proposed mechanism involves TRPM2 (Transient Receptor Potential Melastatin2)-dependent Ca^2+^ dysregulation. These channels, activated by ROS (via ADP-ribose), not only respond to ROS but also amplify it, creating a self-sustaining cycle. Recent studies suggest that TRPM2 activation triggers a cascade of signals from intracellular organelles, enhancing ROS production and affecting cell physiology and viability. This review examines the role of TRPM2 channels in oxidative stress-associated cardiovascular and metabolic diseases. Oxidative stress induces TRPM2-mediated Ca^2+^ influx, leading to lysosomal damage and the release of Zn^2+^ from lysosomal stores to the mitochondria. In mitochondria, Zn^2+^ facilitates electron leakage from respiratory complexes, reducing membrane potential, increasing ROS production, and accelerating mitochondrial degradation. Excess ROS activates PARP1 in the nucleus, releasing ADP-ribose, a TRPM2 agonist, thus perpetuating the cycle. Lysosomes act as Ca^2+^-sensitive signalling platforms, delivering toxic Zn^2+^ signals to mitochondria. This represents a paradigm shift, proposing that the toxic effects of Ca^2+^ on mitochondria are not direct, but are instead mediated by lysosomes and subsequent Zn^2+^ release. This cycle exhibits a ‘domino’ effect, causing sequential and progressive decline in the function of lysosomes, mitochondria, and the nucleus—hallmarks of ageing and oxidative stress-related cardiovascular and metabolic diseases. These insights could lead to new therapeutic strategies for addressing the widespread issue of cardiovascular and metabolic diseases.

## 1. Introduction

Cardiovascular diseases (CVDs), including heart attack, stroke, heart failure, and hypertension, are the leading cause of death globally, accounting for approximately 18 million deaths annually, nearly one-third of all global mortality in 2019. Over 80% of these deaths were due to heart attack and stroke. The prevalence of metabolic diseases, such as Type 2 Diabetes (T2D), hypertension, hyperlipidaemia, metabolic dysfunction-associated steatotic liver disease (MASLD), and obesity, has increased significantly over the past two decades, particularly in low- to low-middle-income countries (cardiovascular diseases (CVDs)) [1]. These conditions are closely interconnected, with metabolic diseases driving the development and progression of CVDs and other chronic illnesses, sharing common risk factors. Collectively, CVDs and metabolic diseases represent a major public health crisis [2], reducing healthspan and lifespan, and imposing substantial economic costs [3]. Despite significant improvements in treatment outcomes, current therapies often involve complex medication regimens (polypharmacy) due to the frequent co-existence of CVDs and metabolic diseases in affected individuals [3]. Therefore, there is an urgent need to deepen our understanding of the fundamental mechanisms underlying these diseases, such as oxidative stress, to address their root causes. This review discusses evidence that while TRPM2 (Transient Receptor Potential Melastatin 2) directly regulates Ca^2+^, it indirectly causes organelle Zn^2+^ dyshomeostasis. We propose that while Ca^2+^ regulation supports physiological signalling, Zn^2+^ dysregulation is a key driver of reactive oxygen species (ROS)-linked non-communicable diseases (NCDs) and ageing.

### 1.1. Oxidative Stress as a Common Pathogenic Feature of Cardiovascular and Metabolic Diseases

Oxidative stress is a prevalent pathogenic feature in cardiovascular and metabolic diseases, arising from a redox imbalance where ROS production overwhelms the body’s antioxidant defences [4,5,6,7,8,9,10,11,12,13,14]. Although antioxidants like vitamins C and E, and glutathione precursors such as N-acetyl cysteine, have shown promise in preclinical trials, they have largely failed in clinical trials [9,12,13,15,16]. This failure is attributed to the dual role of ROS: while excess ROS contribute to pathogenic effects, physiological levels of ROS are crucial for redox signalling, which supports normal health by regulating cell proliferation and differentiation [9,12,13,15,16]. Physiological ROS selectively target signalling proteins, such as enzymes and transcription factors, whereas excess ROS indiscriminately attack biomolecules like DNA, proteins, and lipids, leading to the activation of proinflammatory pathways (e.g., via NF-kB activation) with pathological outcomes [4,5,6,7,8,9,10,11,12,13,14]. Therefore, it is essential to target the mechanisms responsible for excess ROS production rather than attempting to neutralize ROS broadly with antioxidants. This is particularly important given that lifestyle changes (sedentary behaviour, overnutrition, and increased reliance on processed foods) and climate change (pollution) are increasingly driving CVDs and metabolic diseases [2].

Recent studies suggest that many of these diseases share a common signalling mechanism involving the upregulation of pathogenic ROS generation and the associated decline in the structural and functional integrity of organelles, particularly mitochondria and lysosomes, leading to cell dysfunction or death [17]. One mechanism closely linked to ROS upregulation is Ca^2+^ signalling. This review summarizes our current understanding of how ROS upregulate Ca^2+^ signalling, which in turn amplifies ROS production through a mechanism known as ROS-induced ROS production (RIRP), causing progressive organelle damage and cell dysfunction. We will conclude by discussing the potential therapeutic opportunities these recent findings present for the treatment of cardiovascular and metabolic diseases.

### 1.2. ROS-Sensitive Calcium Channels Relevant to Cardiovascular and Metabolic Diseases

Cells express several types of ROS-sensitive Ca^2+^ channels that play crucial roles in cardiovascular and metabolic diseases. These channels are found both at the plasma membrane and within intracellular organelles, including the endoplasmic reticulum (ER). Key channels include the TRP (Transient Receptor Potential) family (TRPC, TRPM2, TRPV1, TRPV4) [18,19,20,21,22], Orai channels [23,24], and ryanodine receptors (RyRs) [25,26], the inositol 1,4,5-trisphosphate receptor (IP3R) [27] which link oxidative stress to Ca^2+^ signalling dysregulation in these diseases [28,29].

TRPC channels are involved in vascular smooth muscle contraction and endothelial dysfunction, processes relevant to hypertension and atherosclerosis [19,21,30]. ROS can modulate TRPV1 and TRPV4 in vasculature, potentially affecting vascular tone and inflammatory responses [31]. Orai channels, the pore-forming subunits of Calcium Release-Activated Calcium (CRAC) channels, mediate store-operated Ca^2+^ entry (SOCE) indirectly through ROS-sensing ER-localized STIM proteins [23,24,29]. These channels are implicated in various cardiovascular diseases, including heart failure and hypertension, as well as metabolic disorders affecting immune cell function and inflammation [23]. RyRs, located in the sarcoplasmic reticulum (SR) of muscle cells and the ER of non-muscle cells, play roles in excitation–contraction coupling in the heart. ROS increase the open probability of these channels, leading to increased Ca^2+^ release from the SR/ER, potentially contributing to arrhythmias and heart failure [25,26].

Among these calcium channels, the TRPM2 channel is of particular interest due to its potent activation by ROS and its implication in numerous NCDs, including neurodegenerative, cardiovascular, and metabolic diseases, as well as cancer [32,33,34,35,36]. Experimental disease models have shown that preventing TRPM2 channel activation mitigates many conditions, including diabetes, obesity, hypertension, atherosclerosis, ischemia/reperfusion (I/R) injury, heart failure, and endothelial dysfunction [37,38] (Table 1). This review analyses the literature to formulate a shared mechanism underlying these diseases, highlighting the central role of ROS activation of the TRPM2 channel, which may explain its association with a wide spectrum of diseases.

### 1.3. ROS Activation of TRPM2

**Figure 1:**
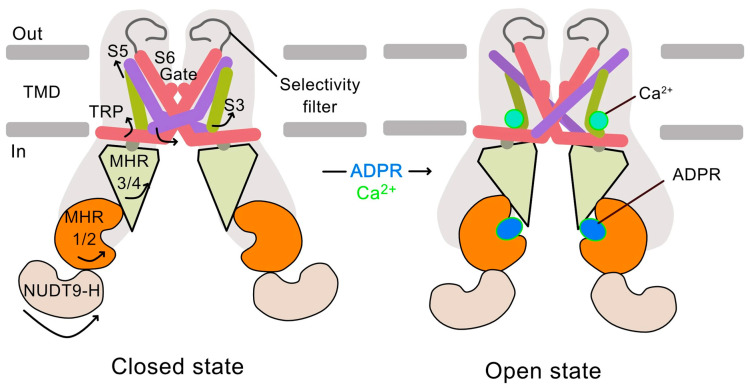
Structure-informed mechanism for TRPM2 activation. A schematic representation of the TRPM2 channel activation mechanism. Two subunits are shown for clarity; key domains linked to channel activation are labelled. The model highlights the C-terminal NUDT9H domain, which contains the binding site for the agonist ADPR, and the S2-S3 loop, which forms the Ca^2+^-binding site. In its closed state, the channel is inactive. Upon binding of ADPR, the NUDT9H domain undergoes a conformational change, priming the channel. The synergistic binding of intracellular Ca^2+^ induces a further rotation and tilt in the transmembrane helices, leading to the opening of the S6 activation gate and allowing Ca^2+^ influx across the plasma membrane. The major movements induced upon binding of ADPR and Ca^2+^ are shown by arrows. Adapted from [41].

Unlike most ROS-sensitive channels, TRPM2 is activated indirectly by ROS through an increase in the intracellular concentration of its primary agonist, ADP-ribose and Ca^2+^ (ADPR) [39,40]. Elevated ROS levels cause DNA damage, including single-strand breaks, which activate the nuclear enzyme poly(ADP-ribose) polymerase 1 (PARP1), a DNA damage sensor. Activated PARP1 consumes NAD^+^ to form poly(ADP-ribose) (PAR) chains on nuclear proteins. These chains are then degraded by poly(ADP-ribose) glycohydrolase (PARG), increasing the intracellular concentration of the TRPM2 agonist, ADPR. ADPR then binds to TRPM2 channels, priming them for activation [33,34]. However, the channels require the synergistic binding of intracellular Ca^2+^ ions to open (Figure 1) [40,41]. Thus, the increased ADPR levels resulting from the ROS-PARP1-PARG signalling axis serve as a crucial link between oxidative stress and TRPM2-mediated calcium influx in various pathological conditions. Supporting this mechanistic link, many diseases associated with PARP1 activation are similar to those linked to TRPM2 overactivation (Table 1).

### 1.4. The TRPM2 Calcium Channel: Structure, Activation, and Function

The TRPM2 ion channel is composed of four identical subunits that assemble to form a tetramer, creating a central ion-conducting pore. Each subunit consists of six transmembrane helices (S1–S6): S1–S4 form the voltage sensor-like domain, while S5–S6 contribute to the pore domain, with the cytoplasmic ends of S6 forming the activation gate [33,34,39,41,42]. The N-terminal domain contains the TRPM homology region (MHR) with four domains (MHR1-4), and the C-terminal domain contains the NUDT9H domain. The NUDT9H domain has a binding site for ADPR, while the Ca^2+^-binding site is located at the S2-S3 loop [41].

Cryo-EM structures of the channel have provided insights into the molecular mechanism by which ADPR and Ca^2+^ act synergistically to open the S6 gate of the TRPM2 channel (Figure 1) [41]. The C-terminal NUDT9H domain folds back to interact extensively with the N-terminal MHR1/2 domains both in *cis* and *trans*. Upon ADPR binding, the NUDT9H and MHR1/2 domains undergo a 27° rigid body rotation, disrupting the trans interaction and priming the channel for opening. Subsequent Ca^2+^ binding induces a 15° rotation in the cytoplasmic domain, a tilt of the TRP helix, and a twist of the pore-facing S6 helix, ultimately opening the S6 gate and allowing Ca^2+^ influx [41].

### 1.5. Sources of ROS Involved in CVDs and Metabolic Diseases

Since ROS activation of the TRPM2 channel is central to most cardiovascular and metabolic diseases, it is essential to review the sources and dynamics of ROS and the mechanisms by which they are amplified in disease conditions. ROS include various reactive oxygen derivatives, such as free radicals (e.g., superoxide, O_2_•^−^; hydroxyl radical, •OH) and non-radicals (e.g., hydrogen peroxide, H_2_O_2_). While O_2_•^−^ and H_2_O_2_ contribute to physiological redox signalling, the highly reactive •OH is primarily implicated in oxidative damage and disease progression. The major producers of ROS in cardiovascular and metabolic diseases are NADPH oxidases (NOXs) and mitochondria, which catalyze the conversion of O_2_ to the superoxide radical O_2_•^−^.

#### 1.5.1. The NOX Family

**Figure 2:**
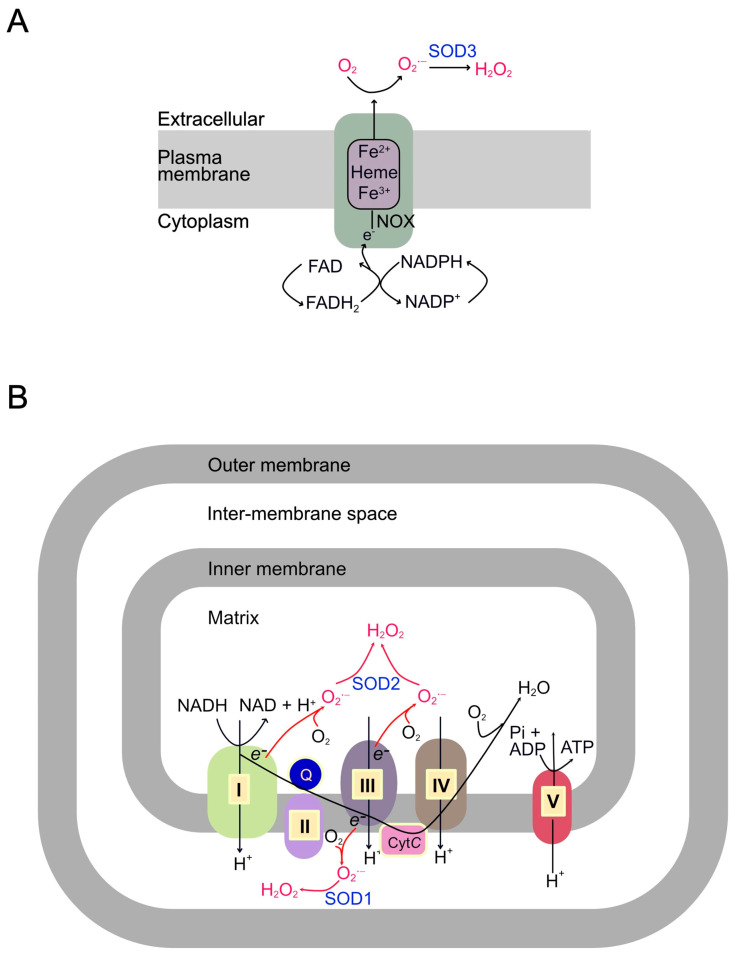
**Sources of cellular ROS.** (**A**) ROS generation at the plasma membrane by NADPH oxidase (NOX). The NOX enzyme complex transfers an electron (e^−^) from cytoplasmic NADPH to extracellular O_2_, producing the superoxide radical (O_2_•^−^). Extracellular superoxide dismutase (SOD3) converts this to hydrogen peroxide (H_2_O_2_), which can enter the cell. (**B**) ROS production from the mitochondrial electron transport chain (ETC). During normal respiration, electrons from NADH are passed along Complexes I-IV to reduce O_2_ to H_2_O. However, electrons can prematurely leak from Complex I and Complex III, reacting with O_2_ to form O_2_•^−^ in the mitochondrial matrix and intermembrane space, respectively (shown in red). This O_2_•^−^ is converted to H_2_O_2_ by SOD2 (matrix) and SOD1 (intermembrane space).

The human NOX family comprises NOX1-5 and the dual oxidases DUOX1-2 [43,44,45]. All NOX enzymes share a core structure with a membrane-embedded domain containing haeme groups and a cytoplasmic dehydrogenase domain with NADPH and FAD-binding sites. NOX enzymes facilitate the transfer of electrons from NADPH via FAD and haeme groups to extracellular O_2_, producing O_2_•^−^ (Figure 2A) [43,44,45,46]. Some NOX isoforms (NOX1 and NOX2) also have several accessory subunits required for their activity, which reside in the cytoplasm and are recruited in response to stimuli [44,45,47]. Extracellular superoxide dismutase 3 (SOD3) converts O_2_•^−^ to H_2_O_2_, which can enter the cell via aquaporins or diffusion [6,43].

Although the primary function of NOXs is regulated ROS generation in phagocyte host defence, extensive evidence implicates NOX enzymes in the pathogenesis of many NCDs, including chronic inflammation, cardiovascular diseases, and metabolic diseases. Different isoforms are expressed in various tissues and implicated in disease [43,44,45,46]. NOX1, NOX2, NOX4, and NOX5 are expressed in vascular cells (endothelium, vascular smooth muscle cells), cardiomyocytes, adipocytes, and pancreatic β-cells [43]. They are activated by various stimuli, including growth factors like angiotensin II, inflammatory cytokines such as TNF-α, and metabolic signals like high glucose, contributing significantly to hypertension, atherosclerosis, cardiac remodelling, insulin resistance, and diabetic complications (Table 1).

Extensive experimental evidence, from genetic knockout animal models to studies on human tissues, confirms the pivotal role of NOX enzymes in the pathology of vascular diseases like hypertension and atherosclerosis (Table 1). Furthermore, their activity is central to fuelling chronic inflammation, contributing to metabolic dysfunction seen in obesity, and promoting the development of insulin resistance by impairing insulin signalling pathways and damaging pancreatic β-cells, making them significant targets for future therapeutic strategies (Table 1).

#### 1.5.2. Mitochondria

Mitochondria are double-membrane organelles with an outer membrane (OMM) and inner membrane (IMM) separated by the intermembrane space (IMS). The IMM encloses the matrix and contains the electron transport chain (ETC), responsible for ATP production and most ROS generation (Figure 2B). Complexes I (NADH–ubiquinone oxidoreductase) and III (ubiquinol–cytochrome *c* oxidoreductase) of the ETC are the major sources of ROS. Under basal conditions, electrons from NADH and FADH_2_ pass along the ETC to O_2_, reducing it to H_2_O at Complex IV. The primary function of this electron transport is to pump protons across the IMM, generating a H^+^ gradient that, together with the electrical gradient, creates an electrochemical potential across the IMM (ΔΨmt). During this transport, electrons can leak prematurely from the complexes into the matrix and IMS, reacting directly with O_2_ to generate superoxide (O_2_•^−^). During oxidative stress, this leakage increases markedly, generating excessive amounts of ROS (mtROS) [9,48,49].

mtROS produced at Complex I is released into the mitochondrial matrix, where it is converted to H_2_O_2_ by SOD2. Due to the impermeability of the IMM to O_2_•^−^ and the need to cross both mitochondrial membranes, it is unlikely that much of Complex I-generated O_2_•^−^ exits into the cytoplasm. Conversely, Complex III releases O_2_•^−^ into the IMS, where it is converted to H_2_O_2_ by SOD1 [50,51]. Some Complex III-generated O_2_•^−^ may exit into the cytoplasm via the outer membrane VDAC (voltage-dependent anion channel), whereas H_2_O_2_ can diffuse out or exit via porins [52,53].

Under certain conditions, such as ischemia–reperfusion (I/R), increased substrate availability and ΔΨmt (hyperpolarization) cause electrons to flow backward from Complex II through Complex I (reverse electron transport, RET), becoming a major source of O_2_•^−^ [11,48,54]. From a therapeutic perspective, determining the relative contributions of forward and reverse electron transport, as well as the individual complexes, to mtROS production may seem important. However, this could be unviable given the interplay between the complexes and their propensity to assemble into super-complexes [55].

### 1.6. Antioxidants

Cells possess both enzymatic and nonenzymatic defence mechanisms to counteract excessive ROS accumulation [5,13,16,48,56,57,58]. Enzymatic mechanisms include superoxide dismutases (SOD1-3), which convert O_2_•^−^ to H_2_O_2_. This H_2_O_2_ is subsequently neutralized by catalase and glutathione peroxidases. Other ROS-metabolizing mechanisms involve peroxiredoxins (Prx1-6) and the thioredoxin system. Nonenzymatic antioxidants, such as glutathione, vitamins C and E, coenzyme Q10, NADPH, and bilirubin, directly neutralize ROS.

### 1.7. TRPM2-Mediated Ca^2+^ Influx Triggers a Self-Perpetuating ROS Amplification Cycle

TRPM2 channels are expressed in a wide range of tissues, playing roles in cytokine secretion and being implicated in temperature and pain sensation under physiological conditions [33,34,35,37,38,59]. However, during oxidative stress, TRPM2 overactivation leads to Ca^2+^ overload, which is linked to several ROS-driven diseases, including cardiovascular and metabolic diseases [33,34,35,37,38]. Paradoxically, TRPM2 overactivation exacerbates ROS production in most cell types examined [60,61,62]. Although there are exceptions [32,63], many studies have shown that the suppression of TRPM2 channels reduces ROS generation [33,34,37,38,60,61,62,64,65]. This unique ability to amplify oxidative stress is central to its role in mediating ROS-induced cellular dysfunction and apoptosis in various NCDs, including cardiovascular and metabolic diseases [33,34,35,37,38].

**Figure 3:**
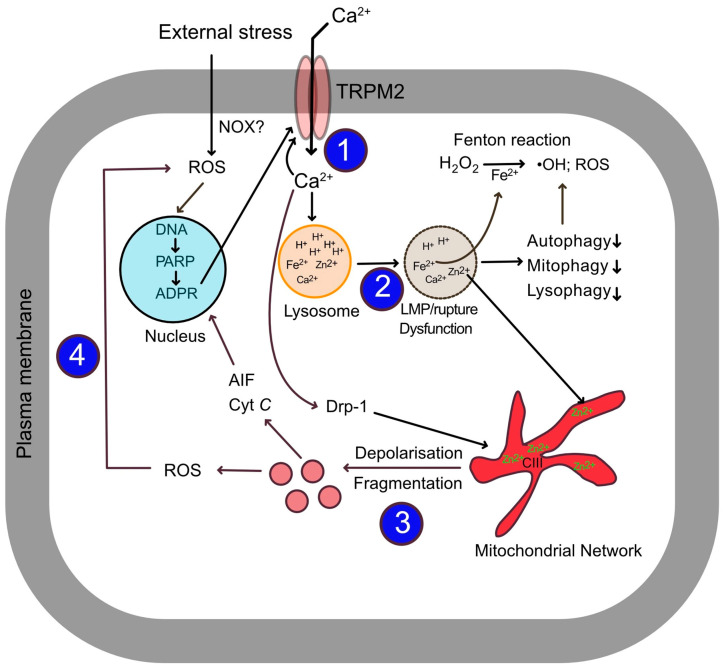
The self-perpetuating cycle of ROS amplification in ocidative stress. 1. Stress Reseponse: External stressors stimulate ROS-mediated ADPR production, priming plasma membrane TRPM2 channels for Ca^2+^-dependent activation, leading to Ca^2+^ influx. 2. Lysosome Dysfunction: Elevated cytoplasmic Ca^2+^ causes lysosomal membrane permeabilization (LMP), resulting in lysosomal dysfunction and the release of sequestered ions, including toxic levels of Fe^2+^ and Zn^2+^, into the cytoplasm. Fe^2+^ catalyzes the production of reactive •OH species. 3. Mitochondrial Dysruption: Zn^2+^ released from lysosomes is taken up by mitochondria, where it targets ETC complexes (primarily Complex III), causing a loss of membrane potential (depolarization), mitochondrial fragmentation (driven by Ca^2+^-dependent Drp-1 recruitment), and a significant increase in mitochondrial ROS (mtROS) production. 4. DNA damage and Feedbackloop: mtROS escape into the cytoplasm and nucleus, causing DNA damage. This activates the nuclear enzyme PARP1, which consumes NAD⁺ to produce the TRPM2 agonist, ADPR. This ADPR then further activates TRPM2 channels at the plasma membrane, returning to restart step 1 of the cycle, perpetuating and amplifying the cycle, leading to progressive organelle damage and cell death. The numbers in the figure refer to the four steps described in the legend.

At the cellular level, Ca^2+^ overload impairs the structural and functional integrity of intracellular organelles, including lysosomes and mitochondria, leading to impaired cellular function or cell death [60,61,62]. Recent studies have revealed that these organelles are not merely targets of Ca^2+^ overload but active participants in generating and relaying signals that can be integrated into a signalling circuit [17]. This proposed circuit generates a ‘domino effect,’ whereby each organelle generates a signal that negatively impacts the next organelle in the circuit. The circuit comprises four organelles (plasma membrane, lysosomes, mitochondria, and the nucleus) and four signals (Ca^2+^, Zn^2+^, ROS, and ADPR) (Figure 3).

External stressors, such as overnutrition, toxins, and pollutants, initiate the signalling cascade, which propagates as follows:

1. Stress activates the TRPM2 channel at the plasma membrane, increasing Ca^2+^ influx.
2. The resulting Ca^2+^ overload leads to lysosomal impairment and redistribution of lysosomal Zn^2+^ to mitochondria.
3. The rise in mitochondrial Zn^2+^ leads to mitochondrial membrane depolarization, breakdown of the branched network, and excessive ROS (mtROS) generation.
4. The mtROS stimulates PARP1 activation in the nucleus, generating ADPR.
5. ADPR feeds back to the plasma membrane TRPM2, perpetuating the cycle and exacerbating progressive organelle damage and cell dysfunction.

In the following sections, we will explain each step of the cycle (Figure 3) in detail, with supporting experimental evidence (where available) in relation to cardiovascular and metabolic diseases.

#### 1.7.1. Step 1: Activation of TRPM2 Channels by External Stressors

Although TRPM2 channels have been implicated in most cardiovascular and metabolic diseases [33,34,37,38] (Table 1), early insights into the underlying signalling mechanisms are only just beginning to emerge (reviewed in [17]). Cellular models of nutrient and oxidative stress have revealed key signals and organelles involved in translating stress signals into cell dysfunction or death [60,61,62]. For example, exposure of endothelial cells to high glucose (diabetic stress) generates TRPM2-dependent signals that cause lysosomal and mitochondrial damage [60], potentially attenuating nitric oxide production required for vasodilation [66]. Similarly, exposure of pancreatic β-cells to free fatty acids (obesity stress) leads to TRPM2-dependent organelle damage and cell death [62]. In both cases, organelle damage and consequent cell dysfunction/death were mitigated by the ROS quencher N-acetyl cysteine, as effectively as TRPM2 inhibition. Furthermore, TRPM2 activation stimulates ROS amplification, creating a self-feedback mechanism that perpetuates TRPM2-driven organelle damage and cell dysfunction/death [17,60,61,62].

These findings confirm the role of the TRPM2 channel as both a sensor and amplifier of oxidative stress. The generation of ROS required for initial TRPM2 activation (i.e., before TRPM2 begins to stimulate ROS production) may depend on the target cell. Although nutrients can stimulate ROS production from NADPH oxidases (NOXs) and mitochondria, the inhibition of NOXs, especially NOX-2, can ameliorate several cardiovascular and metabolic diseases (Table 1), suggesting that NOXs might play a key role in translating nutrient stress into ROS signals required for initial TRPM2 activation. Experimental evidence from β-cells [62], neuroblastoma cells [61], and HEK293 cells conditionally expressing TRPM2 channels [61,62] supports NOX-2 involvement in TRPM2-mediated oxidative damage. Evidence from neuroblastoma cells and HEK293 cells suggests synergistic activation of TRPM2 channels and NOX-2, with a positive feedback relationship between the two proteins (NOX-2 providing ROS for TRPM2 activation and TRPM2 activation providing Ca^2+^ for NOX-2 activation), contributing to the generation of initial Ca^2+^ signals and triggering downstream signalling [17,61]. Given that TRPM2 and NOX-2 are involved in a wide range of NCDs, including some cardiovascular and metabolic diseases (Table 1), this mechanism may be shared by many diseases. It remains to be determined whether any of the other Ca^2+^-dependent NOX isoforms are similarly functionally coupled to TRPM2.

In both studied examples (endothelial cells and β-cells), TRPM2-mediated Ca^2+^ influx caused extensive structural damage to the mitochondrial network, leading to functional decline and increased ROS production—a hallmark of cardiovascular and metabolic diseases, as well as ageing [66,67,68,69,70,71,72,73,74]. In vivo studies have shown that TRPM2 inhibition or genetic knockout increases mitochondrial function, reduces high-fat diet-induced weight gain, and improves insulin sensitivity (Table 1). Furthermore, TRPM2 inhibition ameliorated experimentally induced hypertension. Given the interconnected nature of cardiovascular and metabolic diseases, it is likely that the TRPM2-driven signalling mechanism is conserved between these diseases. However, further cell biological studies are required to test this possibility in different disease models. Nevertheless, the two conditions examined thus far—diabetic stress and obesity stress—are major drivers of many diseases, accounting for the majority of premature deaths globally [1,75]. We believe that TRPM2 could be a potential target for developing broad-spectrum therapeutics against the growing number of NCDs.

#### 1.7.2. Step 2: TRPM2-Mediated Rise in Cytoplasmic Ca^2+^ Targets Lysosomes

##### Ca^2+^ First Targets Lysosomes

Although the long-held view is that cytoplasmic Ca^2+^ rise directly targets mitochondria, recent findings suggest an intermediate obligatory step involving lysosomes and Zn^2+^ signals [60,61,62]. High glucose-induced, TRPM2-mediated Ca^2+^ rise leads to a decline in lysosomal function (evidenced by reduced uptake of the pH-dependent LysoTracker dye) and structural damage (measured by the release of cathepsins into the cytoplasm) [60]. This damage could result from lysosomal membrane permeabilization (LMP) or full rupture of the membrane. These lysosomal effects are accompanied by the translocation of mitotoxic Zn^2+^ signals to mitochondria. Sequestration of Zn^2+^ signals with Zn^2+^ chelators (TPEN and clioquinol) protected cells from high glucose-induced mitochondrial damage [60]. Similar observations were made with HEK293 cells engineered to express TRPM2 channels (but not with HEK293 cells lacking TRPM2 expression) upon exposure to H_2_O_2_ stress and neuroblastoma cells exposed to the Parkinsonian toxin MPP^+^ (1-methyl-4-phenylpyridinium) [60,61]. Additionally, directly raising intracellular Ca^2+^ with a calcium ionophore recapitulated the lysosomal and mitochondrial effects mediated by TRPM2 activation [60]. Thus, Ca^2+^-induced lysosomal Zn^2+^ transfer is a necessary upstream event in oxidative stress-induced mitochondrial damage, previously attributed to the direct effect of Ca^2+^.

Lysosomal Functional Decline, Impaired Autophagy, and ROS Production: Lysosomal activities, including the fusion of autophagosomes with lysosomes (lysoautophagy), are critically dependent on the lysosomal H^+^ gradient [76,77,78,79]. This gradient is primarily maintained by the coordinated action of vacuolar ATPase (v-ATPase) and the CLC-7 chloride/H^+^ antiporter. The v-ATPase pumps H^+^ from the cytoplasm into the lysosomal lumen, while the CLC-7 antiporter provides the counter ion, Cl^−^, to sustain electroneutrality. Additionally, lysosomes express TMEM175, a K^+^ channel that functions as a H^+^ leak channel, contributing to lysosomal acidity [76,77,78,79]. The importance of TMEM175 for lysosomal function was highlighted by the discovery that the gene encoding this channel is a significant risk factor for Parkinson’s disease. The depletion of the TMEM175 gene results in unstable lysosomal pH, impairing autophagy and leading to excessive ROS production from uncleared toxic waste, including dysfunctional mitochondria and protein aggregates [80,81]. While the precise mechanisms by which Ca^2+^ affects lysosomal pH regulators remain unclear, there is some evidence that elevated Ca^2+^ levels can cause the disassembly of v-ATPase, resulting in its functional loss [82]. This functional decline of lysosomes is a known cause of impaired autophagy and increased ROS production [76,77,78,79].

##### Decline in Lysosomal Numbers, ROS Production and Cell Death

As mentioned above, besides affecting lysosomal pH, Ca^2+^ impairs the structural integrity of lysosomes, leading to a decline in their number. Since lysosomes perform crucial roles beyond the degradation and recycling of cellular waste, including plasma membrane repair, nutrient signalling, and ion homeostasis, the quantity and quality of lysosomes are meticulously maintained through cycles of fission and fusion, lysoautophagy, and biogenesis [78,83]. Fission and fusion enable the segregation of damaged lysosomes from functional ones, while lysoautophagy degrades the dysfunctional lysosomes. Lost lysosomes are replaced through the synthesis of new lysosomes, with lysosomal TRPML-1 channels playing a crucial role. TRPML-1 senses the decrease in lysosomal number, promotes Ca^2+^ release, and facilitates the nuclear translocation of TFEB [84,85,86,87]. TFEB drives the transcription of genes encoding lysosomal proteins involved in lysosomal stability and acidification, replenishing the lost lysosomes. Careful coordination of these mechanisms ensures the functional integrity and density of lysosomes required for the health of an organism [77,78]. Further studies are needed to determine whether Ca^2+^ plays a role in any of these regulatory processes.

Besides H^+^, lysosomes contain high concentrations of free calcium, iron, zinc ions, and hydrolases (e.g., cathepsins). LMP and lysosomal rupture release these lysosomal contents into the cytoplasm. Cathepsins and other proteases trigger apoptosis (lysosome-mediated apoptosis) by hydrolysing cytoplasmic proteins [88,89,90]. Fe^2+^ generates highly reactive •OH radicals from cytoplasmic H_2_O_2_ through the Fenton reaction, triggering oxidative damage. Although redox-inert, Zn^2+^ indirectly contributes to ROS production [15,16]. A decline in lysosomal numbers would also increase ROS production due to the reduced capacity to remove protein aggregates and damaged mitochondria [76,77,78,79].

##### Lysosomal Dyshomeostasis in CVDs and Metabolic Diseases

Although the mechanisms by which a rise in cytoplasmic Ca^2+^ leads to lysosomal damage are not fully understood, substantial evidence links defective autophagy to cardiovascular and metabolic diseases [76,78,88,89,91,92,93,94]. Impaired autophagy not only increases oxidative stress but also promotes chronic inflammation (e.g., NLRP3 inflammasome activation) and metabolic dysregulation [36,77,95,96]. This explains the beneficial effects of ROS quenchers and autophagy inducers (e.g., rapamycin, an mTOR inhibitor; metformin, an AMPK activator) observed in preclinical studies using models of cardiovascular and metabolic diseases [13,79,88]. Chronic oxidative stress causes lysosomal dysfunction and a decline in lysosomal density, making the restoration of lysosomal function and density crucial. Current strategies include using activators of v-ATPase and TMEM-175 to restore lysosomal function and stimulating biogenesis with activators of TRPML1 or TFEB to increase lysosomal numbers [79,88].

New findings suggest TRPM2 as an additional target [17,60,61]. Unlike current restorative strategies, inhibitors of TRPM2 channels could prevent lysosomal dysfunction and loss, thereby ameliorating oxidative stress and autophagy impairment. Supporting this idea, Zhong et al. demonstrated that *Trpm2* knockout prevents ROS-induced NLRP3 inflammasome activation, while other studies have reported the restoration of autophagy through TRPM2 inhibition [36].

#### 1.7.3. Step 3: A Paradigm Shift–Mitochondrial Damage Is Driven by Lysosomal Zn^2+^, Not Directly by Ca^2+^

##### Ca^2+^-Induced Rise in Mitochondrial Zn^2+^ as the Primary Driver of Mitochondrial Damage

As mentioned in the previous section, ROS activation of TRPM2 channels induces Ca^2+^-mediated lysosomal impairment, leading to a rise in mitochondrial Zn^2+^, breakdown of the mitochondrial network, loss of membrane potential (ΔΨmt), and increased production of mtROS. Notably, these mitochondrial events are mediated almost exclusively by Zn^2+^ acquired from lysosomal damage in all cellular disease models studied thus far [17,60,61,62,73]. Importantly, raising intracellular Ca^2+^ directly with a calcium ionophore mimicked the effects of TRPM2-mediated Ca^2+^ rise on organelle damage, but the ionophore-induced damage was fully abolished by a Zn^2+^ chelator [60]. These findings suggest that Zn^2+^ acts downstream of Ca^2+^, indicating that the Ca^2+^ effects on mitochondria, previously thought to be direct, are mediated by Zn^2+^.

Although there is no direct in vivo evidence supporting Zn^2+^ acting downstream of Ca^2+^, increases in intracellular Zn^2+^, including within mitochondria, have been reported in several models of myocardial and neuronal ischemia–reperfusion (IR) injury [17,60,61,62,97,98,99,100,101,102,103,104] (Table 1). Furthermore, the Zn^2+^ chelator TPEN reduced infarct size and rescued neuronal death following a 90 min middle cerebral artery occlusion in a rat model of stroke and in isolated rat hearts subjected to IR injury [105] (Table 1). Importantly, knockout of the TRPM2 channel prevented the rise in cellular Zn^2+^, protecting mice from pyramidal neuronal cell death and memory impairment in a transient global ischemia model [100]. Additionally, TRPM2 deficiency prevented increases in intracellular Zn^2+^, ROS, and neuronal cell death in hippocampal brain slices subjected to oxygen-glucose deprivation [100]. These in vivo findings support the idea that oxidative stress causes neuronal cell death by inducing a TRPM2 (Ca^2+^)-induced rise in mitochondrial Zn^2+^.

Several studies have suggested a role for Zn^2+^ in many diseases, including cardiovascular, neuronal, and metabolic diseases (Table 1). However, this role was largely ignored by the scientific community due to the lack of a mechanistic explanation. Researchers have warned that the effects attributed to Ca^2+^ alone could, at least in part, be due to Zn^2+^ because probes commonly used to determine the roles of Ca^2+^, including BAPTA and its derivatives, have much greater (>10-fold) affinity for Zn^2+^ than for Ca^2+^ [106,107]. The mechanistic explanation presented here helps reconcile these controversies by invoking Zn^2+^ as the downstream signal of Ca^2+^. More importantly, it highlights the importance of investigating the essential roles that organelles play in coordinating the effects of Ca^2+^ and Zn^2+^.

While lysosomal damage is accompanied by a rise in mitochondrial Zn^2+^, how lysosomal Zn^2+^ is redistributed to the mitochondrial matrix is not clear. The mitochondrial uniporter is a potential candidate, but there is no direct evidence [104,108]. It is plausible that lysosome–mitochondria contact sites [87,94,109,110,111], which are implicated in mitochondrial fission and exchanges of ions and metabolites, offer avenues for Zn^2+^ transfer before being sequestered by cytoplasmic protein buffers. Studies of genetic mutations linked to Parkinson’s disease provided evidence for the mito-toxic role of lysosomal Zn^2+^ [112,113]. These studies demonstrated that genetic mutations in *PARK9* (encoding ATP13A2) reduce lysosomal Zn^2+^ uptake in patient-derived cells while increasing mitochondrial Zn^2+^, resulting in mitochondrial damage. These effects are similar to the effects of nutrient, H_2_O_2_, or toxin stress on other cell types [17,60,61], suggesting the wider pathophysiological significance of lysosomal Zn^2+^.

##### *Z*n^2+^ Targets Mitochondrial Complexes, Primarily Complex III, to Cause Loss of ΔΨmt and Exacerbate ROS Production

Given the evidence that Zn^2+^, in addition to Ca^2+^, can stimulate mitochondrial ROS production, we consider the relative contributions of Ca^2+^ and Zn^2+^ to mitochondrial ROS production. It is widely acknowledged that Ca^2+^ overload causes the loss of ΔΨmt (and the associated bioenergetic failure), increasing ROS production by targeting many ROS-generating sites in the mitochondria, including Complexes I and III, the major producers of ROS [5,7,11,48,114,115]. However, the biochemical and molecular basis for how Ca^2+^ stimulates ROS production is unclear. It is speculated that Ca^2+^ induces protein conformational changes to stimulate ROS production. By contrast, structure–function studies have identified distinct Zn^2+^-binding sites on Complexes I and III, with Zn^2+^ binding linked to ROS production. There is a significant difference in their affinity for Zn^2+^, with Complex III having a greater affinity than Complex I [114,115]. Given that mitochondrial Zn^2+^ concentration is extremely low [116], it is unlikely that Zn^2+^ would contribute to mtROS production under physiological conditions. Any ROS produced during normal conditions and mild stress is likely due to natural electron leak and Ca^2+^-stimulated electron escape, contributing to physiological signalling.

During oxidative stress, however, Ca^2+^-induced translocation of lysosomal Zn^2+^ to mitochondria could elevate Zn^2+^ concentration high enough to exacerbate electron leak from the complexes. Currently, there is no quantitative data on the magnitude of mitochondrial Zn^2+^ increase, but given that Complex III has a much greater affinity for Zn^2+^ than Complex I, Complex III [114,115] is arguably the major target for Zn^2+^. Supporting this possibility, studies with neuronal and non-neuronal cell lines demonstrated that quenching of Complex III-generated electrons with S3QEL (a chemical capable of selectively quenching electrons generated at Complex III) [117] abolished ROS production and cell death [61]. In vivo studies using disease models provide additional support. Genetic deletion of functionally important Complex III subunits in neurons attenuates ROS-mediated cell death and motor deficits in a model of Alzheimer’s disease [118]. Furthermore, genetic knockout of Complex III in pancreatic β-cells caused early hyperglycaemia, glucose intolerance, and loss of glucose-stimulated insulin secretion [119]. Together, these findings suggest that elevation of mitochondrial Zn^2+^ leads to increased ROS production from Complex III, primarily impacting mitochondrial health.

##### Zn^2+^ Plays a Major Role in Mitochondrial Fragmentation 

Concomitant with the increase in ROS production, the rise in mitochondrial Zn^2+^ causes the breakdown of the mitochondrial network [60,62], a hallmark of most NCDs and ageing, as well as numerous other diseases [67,68,69,70,72,120,121]. Earlier studies have implicated Ca^2+^ as the key driver of mitochondrial fission [122]. However, the finding that chelation of Zn^2+^ abolishes oxidative stress-induced mitochondrial fission [60,62] warrants a fresh review of the relative roles of Ca^2+^ and Zn^2+^ in mitochondrial dynamics, which refers to the continuous and tightly regulated processes of fusion, fission, transport, and mitophagy that govern mitochondrial shape, size, distribution, and quality within a cell.

Mitochondrial fission, mediated by Drp-1 (dynamin-related protein-1), enables the segregation and removal of dysfunctional mitochondrial components through mitophagy. Conversely, mitochondrial fusion, catalyzed by Mfn1, Mfn2, and Opa1, facilitates the merging of functional mitochondrial portions, contributing to the expansion and interconnectivity of healthy mitochondrial networks. This fusion is essential for maintaining mitochondrial health and restoring functionality after stress or damage. An increase in fission relative to fusion is a common feature of most NCDs [67,68,69,70,72,120,121].

We will consider how the rise in cytoplasmic Ca^2+^ and mitochondrial Zn^2+^ impacts Drp-1-mediated mitochondrial fission. During oxidative stress, mitochondria lose their ΔΨmt, triggering Drp-1 recruitment from the cytoplasm [68,72,123]. Given the evidence presented above, we suggest that Zn^2+^-induced loss of ΔΨmt primes mitochondria for Drp-1 recruitment, while Ca^2+^ promotes Drp-1 recruitment through permissive phosphorylation regulated by calcium-calmodulin-dependent phosphatase calcineurin (CaN) [122]. This could mean that a modest increase in cytoplasmic Ca^2+^ would promote Drp-1 recruitment to delete naturally worn-out portions of the mitochondrial network, thereby restoring mitochondrial health. During oxidative stress, Zn^2+^ joins Ca^2+^ to increase Drp-1 recruitment, exacerbating mitochondrial fragmentation. Thus, while physiological fluctuations in Ca^2+^ support mitochondrial health, Ca^2+^ overload via Zn^2+^ causes mitochondrial damage.

In preclinical disease models, inhibition of Drp-1 demonstrated protective effects against cardiovascular diseases, including ischemia–reperfusion (I/R) injury to the brain [122,124] and heart [125], as well as metabolic diseases [126], improving insulin sensitivity, decreasing inflammation, and reducing body weight in diet-induced obesity models. These protective roles were linked to the ability of Drp-1 inhibition to prevent excessive ROS production. Zn^2+^ chelation has similar protective roles in several of these diseases [17,60,61,62,104], consistent with the proposed role of Zn^2+^ in mitochondrial recruitment of Drp-1. The role of Zn^2+^ is better established in neurodegenerative diseases, including Parkinson’s and Alzheimer’s diseases, with elevated levels of Zn^2+^ found in patient brains.

##### Therapeutic Potential of TRPM2-Ca^2+^-Zn^2+^-Mediated Mechanism 

The TRPM2-Ca^2+^-Zn^2+^ mediated mechanism may offer new therapeutic opportunities to the expanding arsenal of mitochondrial medicines. Drugs in development target core issues affecting mitochondrial structure and function, such as mitochondrial fission (Drp-1 inhibitors), mitophagy (rapamycin, metformin), excess ROS (mitochondria-targeted antioxidants like MitoQ), and function (NAD^+^ supply) [12,13,16,127]. While these approaches have shown promise in preclinical studies, translation into clinical practice has proved challenging. Given that inhibition of the TRPM2 channel and Zn^2+^ chelation rescues the structural and functional integrity of mitochondria in various oxidative stress-linked disease models, including cardiovascular and metabolic diseases, we believe that this mechanism offers new opportunities for a ‘common’ mitochondrial medicine for a wide range of diseases, with potential for prophylactic use.

While several TRPM2 inhibitors targeting the channel pore (e.g., N-(p-amylcinnamoyl)anthranilic acid, clotrimazole, econazone, flufenamic acid) or the ADPR-binding site (e.g., 8Br-ADPR) have proven useful in determining the physiological and pathophysiological roles of TRPM2 channels, their therapeutic usefulness is hampered by the lack of specificity [37]. Pore-blocking antibodies [128] likely overcome this limitation of poor selectivity. Likewise, multiple studies have demonstrated the ability of TPEN, a high affinity Zn^2+^ chelator, in rescuing oxidative stress-induced CVDs and metabolic diseases in preclinical studies (see Table 1). Clioquinol, a repurposed drug, and PBT compounds have been explored for their therapeutic potential mainly in relation to neurodegenerative diseases [129,130], but clioquinol demonstrated protective function in preclinical models of diabetes [131] and stroke [132].

#### 1.7.4. Step 4: Mitochondrial ROS Generates ADPR from the Nucleus for Feedback Activation of TRPM2, Perpetuating the Cycle

ROS generated from damaged mitochondria escape into the cytoplasm, affecting DNA, proteins, and lipids. A key target of ROS is nuclear DNA. ROS induces DNA strand breaks (primarily single-strand breaks), which are promptly detected by PARP1. The binding of PARP1 to DNA breaks causes a conformational change in the enzyme, leading to its extensive activation [133]. As explained earlier, PARP1, together with PARG, initiates DNA repair, but during the process generates ADPR, a potent activator of the TRPM2 channel. Thus, the nucleus serves as the third organelle in the cycle, contributing to the perpetuation of the ROS cycle through feedback activation of the TRPM2 channel. Remarkably inhibition of PARP1 has beneficial effects similar to TRPM2 inhibition in NCDs (Table 1). The propagation of this vicious cycle causes progressive damage to the organelles (lysosomes, mitochondria, and the nucleus) participating in the cycle (Figure 3). While in a normal cell, these organelles cooperate to support cellular homeostasis, during oxidative stress, they participate in a cyclical process leading to self-destruction and eventual cellular dysfunction and/or death.

## 2. Outstanding Questions

While this review synthesizes evidence into a coherent cyclical mechanism, it also highlights several critical gaps in our knowledge that represent exciting avenues for future research. First, one key area is the lysosome–mitochondria interface: understanding how Zn^2+^ is mobilized from damaged lysosomes and specifically transported to mitochondria is crucial. This transfer might be mediated by direct lysosome–mitochondria membrane contact sites or involve specific cytoplasmic chaperones or transporters. Second, quantifying the dynamics of this Zn^2+^ flux is a key next step, as this is an important determinant of ROS amplification. Third, an important aspect is the molecular basis of lysosomal damage. Identifying the precise molecular targets of the initial Ca^2+^ influx that leads to lysosomal membrane permeabilization (LMP) is essential, including the channels, pumps, or structural proteins on the lysosomal membrane affected by Ca^2+^ overload. Fourth, is there a relationship between circadian rhythm and the proposed cycle? Circadian rhythm is being increasingly recognized as a regulator of ROS homeostasis, for instance through regulation of antioxidant enzymes [134,135]. Furthermore, PARP1, regulates the key components of circadian clock such as CLOCK, influencing the expression of clock-controlled genes associated with the development of CVDs and metabolic diseases [135,136,137]. These findings raise the question of whether TRPM2 expression is regulated by circadian rhythm. Fifth, understanding the specificity and context of this TRPM2-driven cycle is vital. Does it operate identically in all cell types, such as endothelial cells, cardiomyocytes, neurons, and pancreatic β-cells, or are there tissue-specific variations in the relative importance of each component (NOX, TRPM2, PARP1)? This context-dependency is crucial for developing targeted therapies. Sixth, the role of this cycle in physiological versus pathological ageing needs exploration. The hallmarks of this cycle—mitochondrial dysfunction, ROS production, organelle decline—are also the hallmarks of ageing. Investigating to what extent low-level, chronic activation of this cycle contributes to the normal ageing process and at what threshold it transitions into the overt pathology seen in NCDs is important. Finally, therapeutic selectivity poses a significant challenge. Given the physiological roles of TRPM2 in the immune system and temperature sensation, understanding the potential on-target side effects of long-term systemic TRPM2 inhibition is crucial. Developing inhibitors with greater selectivity for pathologically over-activated channels or creating targeted delivery systems will be essential.

**Table 1:** Diseases and the various players invloved in TRPM2-driven signalling cycle.

Cardiovascular Diseases					
Disease	TRPM2	NOX	Zinc Involvement	Mitochondrial ROS	PARP1
**Ischaemia-** **Reperfusion: Stroke**	Chemical inhibition or genetic KO of TRPM2 in male mice subjected to I/R injury: ↓Neuronal cell death ↓Infarct size ↓Memory loss [100, 138].	NOX2 KO in mice subjected to I/R injury: Slows the progression of infarct development but does not prevent overall brain damage [139].	↑Zn^2+^ levels in the brain in TBI, but not in TRPM2 KO mice [100]. Zn^2+^ chelation (TPEN, Ca-EDTA) in rodent model: ↓Infarct size [104,140,141].	I/R injury mouse model: ↑Mitochondrial ROS in hippocampus. Scavenging mito-ROS with MitoQ: ↓Hippocampal damage [142]	PARP1 KO in mice: Protects against I/R injury [143].
**Ischaemia-** **Reperfusion: Heart attack**	Chemical inhibition or genetic KO of TRPM2 in male mice subjected to I/R injury: ↓Infarct size ↓Inflammation ↑Cardiac outcome [144].	NOX2 KO in mice subjected to I/R injury: ↓Infarct size [145].	Zn^2+^ chelation (TPEN) in rat: ↓Infarct area in rat hearts during I/R injury [146]. ↑Myocardial recovery in isolated hearts, reducing tissue damage during ischemia (ex vivo model) [147].	Scavenging mito-ROS with MitoQ in I/R injury rat model: ↓Heart dysfunction ↓Mitochondrial damage ↓Cell death [148].	Chemical inhibition of PARP1 in mice subjected to I/R injury: ↓Infarct size ↓Inflammation ↑Cardiac function [149].
**Atherosclerosis**	TRPM2 KO in Apoe-/- mice slows AS progression [150]. TRPM2 KO and KD in EC: ↓Mitochondrial dysfunction and damage [60].	NOX2 KO in Apoe/-e mice: ↓Plaque formation due to NOX2 depletion in macrophages and vessel wall cells [151].	Zn^2+^ levels elevated in advanced human atherosclerotic lesions [152]. Excess mitochondrial Zn^2+^ causes its fragmentation in EC [60].	Scavenging ROS with MitoQ in Apoe/-e mice: ↓Plaques [153].	PARP1 chemical inhibition or KO in Apoe/-e mice: ↓Plaque formation ↓ Progression of AS [154].
**Hypertension**	Patient-derived VSMC: TRPM2 inhibition (siRNA/chemical) reduced Ang II-induced Ca2+ influx. Hypertensive LinA3 mouse model: TRPM2 inhibitors reverse hypertension-associated hypercontractility of mesenteric arteries [155]. TRPM2 activation in EC: ↑Endothelial barrier dysfunction [156]. ↑EC dysfunction [157].	Rodent models: Ang II-induced nitric oxide production rescued by NOX inhibition [158]. NOX1 KO in mice: ↓Ang II-induced hypertension ↓Vascular ROS and remodelling [159].	Endothelial cells: High glucose causes Zn^2+^ dependent mitochondrial damage/dysfunction [60], decreasing NO bioavailability [66].	Ang II-induced and DOCA salt hypertension mouse models: Mito-ROS scavenger (Mito-TEMPO) caused ↑NO bioavailability ↓Blood pressure [160].	Patient-derived VSMC: PARP1 upregulated by Ang II [155]. PARP1 activation in EC: ↑Cell death [161].
**Metabolic Diseases**					
**Disease**	**TRPM2**	**NOX**	**Zinc**	**Mitochondrial ROS**	**PARP1**
**Type 2 diabetes**	TRPM2 KO in mice: ↑Insulin sensitivity ↑Resistance to diet-induced obesity ↑Glucose metabolism ↓Obesity-mediated inflammation [162]. Chemical inhibition or RNAi silencing of TRPM2 in pancreatic β-cells prevents FFA induced: ROS increase, Mitochondrial damage, and Cell death [62].	NOX2 KO in mice: ↑GSIS ↓ROS production [163]. Pancreatic β-cells/islets exposed to FFA: ↓ROS [62].	Zn^2+^ chelation (TPEN): ↓FFA-induced β-cell death [62]. Overexpression of *hZnT8*: ↑Pancreatic Zn^2+^ ↓Insulin and glucose tolerance [164].	Excess nutrition: ↑mtROS production ↑Insulin resistance ↑β-cell dysfunction [165]. Complex III KO mice: Early hyperglycaemia ↑Glucose intolerance ↓GSIS [119].	PARP1 inhibition in diabetic models: ↑Insulin sensitivity ↓Vascular damage [166]. PARP1 inhibitor (PJ34): ↓Pancreatic β-cell death [62].
**Insulin resistance**	TRPM2 KO in obese mice: ↓Insulin resistance in EC [157], skeletal muscle, adipose, heart [162].	NOX2 KO in obese mice: ↓Insulin resistance ↓Superoxide [167]. NOX2 inhibition (chemical and siRNA) in IR -/- mice: ↓Superoxide ↑Vascular function [168].	Chronic high-dose zinc in mice: ↓Glucose tolerance ↑Insulin resistance [169].	Azoxystrobin inhibition of Complex III-generated mtROS production in HFD mice: ↑Glucose tolerance ↑Insulin sensitivity ↓Body weight ↓Liver fat ↑Mitochondrial function [170].	PARP-1 inhibition or KO In HFD mice: ↑Glucose tolerance ↑Insulin sensitivity ↓Weight gain [171].

Abbreviations: ADPR (ADP-ribose), Ang II (Angiotensin II), AS (Atherosclerosis), DOCA (Deoxycorticosterone Acetate), EC (Endothelial Cells), FFA (Free Fatty Acids), GSIS (glucose stimulated insulin secretion), HFD (high fat diet) I/R (Ischemia-Reperfusion), KD (Knockdown), KO (Knockout), mtROS (Mitochondrial ROS), NO (Nitric oxide), OS (Oxidative stress), PARP (Poly(ADP-ribose) polymerase), PJ34 (N-[2-(4-Pyridinyl)-1H-indol-3-yl]methanesulfonamide), TPEN (N,N,N′,N′-Tetrakis(2-pyridylmethyl)ethylenediamine), TBI (Traumatic brain injury), VSMCs (Vascular smooth muscle cells). ↑ (increase/stimulation/worsening of pathology); ↓ (decrease/inhibition/amelioration of pathology).

## 3. Conclusions

Cardiovascular and metabolic diseases, driven by common risk factors like oxidative stress and ageing, represent a monumental global health challenge. Current therapeutic strategies often involve polypharmacy to manage disparate symptoms, highlighting the urgent need for treatments that address the fundamental root causes. This review proposes a unifying mechanism that links these diseases: a self-perpetuating, multi-organelle ‘vicious cycle’ orchestrated by the TRPM2 channel.

We have outlined a four-step ‘domino effect’ where oxidative stress-induced TRPM2 activation and Ca^2+^ influx are not the end of the story, but the beginning. This initial signal triggers lysosomal damage, which in turn unleashes toxic Zn^2+^ signals that cripple mitochondria, leading to amplified ROS production. This mitochondrial ROS then damages nuclear DNA, activating PARP1 to generate more of the TRPM2 agonist ADPR, thus perpetuating the cycle. This model represents a paradigm shift, repositioning lysosomes and Zn^2+^ dyshomeostasis as critical intermediaries between the initial Ca^2+^ signal and subsequent mitochondrial collapse—a role previously attributed almost exclusively to Ca^2+^ overload itself.

Interrupting this cycle presents a powerful and novel therapeutic strategy. Targeting TRPM2, the primary entry point and ‘master switch’ of this cascade, could be a uniquely effective approach. A single TRPM2 inhibitor could theoretically attenuate oxidative stress, rescue mitochondrial structure and function, and protect lysosomal integrity simultaneously. This offers the potential to develop a ‘common’ therapeutic capable of treating a wide spectrum of NCDs, moving beyond single-disease management. Such a drug could even have prophylactic potential for individuals at high risk. By framing TRPM2 as the central node in a network of organelle failure, we can better understand the shared pathology of NCDs and ageing, and open new doors for developing therapies that restore cellular homeostasis and extend healthspan.

## Data Availability

No new data were created or analyzed in this study.

## Abbreviations

ADPR (ADP-ribose), AMPK (AMP-activated protein kinase), β-cell (Beta cell), CVDs (Cardiovascular diseases), ΔΨmt (Mitochondrial membrane potential), Drp-1 (Dynamin-related protein 1), DUOX (Dual oxidase), EC (Endothelial cell), ER (Endoplasmic reticulum), ETC (Electron transport chain), GSIS (Glucose-stimulated insulin secretion), IMM (Inner mitochondrial membrane), IMS (Intermembrane space), IP3R (Inositol 1,4,5-trisphosphate receptor), LMP (Lysosomal membrane permeabilization), MHR (Melastatin homology region), mtROS (Mitochondrial reactive oxygen species), MitoQ (Mitochondria-targeted antioxidant ubiquinone), NADH (Nicotinamide adenine dinucleotide, reduced form), NADPH (Nicotinamide adenine dinucleotide phosphate, reduced), NCDs (Non-communicable diseases), NO (Nitric oxide), NOX (NADPH oxidase), NUDT9H (Nudix-type motif 9 homologue), O_2_•^−^ (Superoxide radical), OMM (Outer mitochondrial membrane), OS (Oxidative stress), PAR (Poly(ADP-ribose)), PARP1 (Poly(ADP-ribose) polymerase 1), PARG (Poly(ADP-ribose) glycohydrolase), PJ34 (N-[2-(4-Pyridinyl)-1H-indol-3-yl]methanesulfonamide), Prx (Peroxiredoxin), RET (Reverse electron transport), ROS (Reactive oxygen species), RyRs (Ryanodine receptors), SOD (Superoxide dismutase), SOCE (Store-operated calcium entry), TFEB (Transcription factor EB), TMEM175 (Transmembrane protein 175), TPEN (N,N,N′,N′-Tetrakis(2-pyridylmethyl)ethylenediamine), TRP (Transient receptor potential), TRPM2 (TRP melastatin 2), TRPML-1 (TRP mucolipin-1), and v-ATPase (Vacuolar ATPase).
